# The Relationships Among Testosterone, Cortisol, and Cognitive Control of Emotion as Underlying Mechanisms of Emotional Intelligence of 10- to 11-Year-Old Children

**DOI:** 10.3389/fnbeh.2019.00273

**Published:** 2019-12-17

**Authors:** Tongran Liu, Danfeng Li, Fangfang Shangguan, Jiannong Shi

**Affiliations:** ^1^CAS Key Laboratory of Behavioral Science, Institute of Psychology, Chinese Academy of Sciences, Beijing, China; ^2^Department of Psychology, University of Chinese Academy of Sciences, Beijing, China; ^3^Beijing Key Laboratory of Learning and Cognition, School of Psychology, Capital Normal University, Beijing, China

**Keywords:** emotional conflict control, cortisol, testosterone, emotional intelligence, preadolescence, event-related potentials

## Abstract

Emotional intelligence is an important factor contributing to social adaptation. The current study investigated how salivary testosterone (T) and cortisol (C) levels, cognitive control of emotional conflict processing were associated with children’s emotional intelligence (EI). Thirty-four 10- to 11-year-old children were enrolled and instructed to complete questionnaires on emotional intelligence as well as empirical tasks of emotional flanker and Stroop with event-related potential (ERP) recordings. Saliva collection took place on another day without ERP tasks. Results showed that lower T and C levels were associated with higher accuracy in emotional conflict tasks, as well as better emotional intelligence (managing self emotions). In the Stroop task, higher T/C ratios were associated with greater congruency effects of N2 latencies, and lower cortisol levels correlated with stronger slow potential activities (SP). For girls, the correlation between cortisol and emotional utilization was mediated by the SP amplitudes on fearful conflicts in the flanker task (95% CI: −8.64, −0.54, *p* < 0.050). In conclusion, the current study found the relationship between cortisol and an emotional intelligence ability, emotional utilization, might be mediated by brain activities during emotional conflict resolution processing (SP responses) in preadolescent girls. Future studies could further investigate testosterone-cortisol interaction and its relation with cognitive control of emotion as underlying mechanisms of emotional intelligence.

## Introduction

The perception, processing, regulation, and utilization of emotional information is well-known as emotional intelligence (EI) (Nelis et al., 2009), which intrinsically includes self-control of emotions (Davis and Rachel, 2016). EI is another phrase for emotional competence (Mayer and Salovey, 1993), which is critical for solving problems like conflict. There is a lot of evidence suggesting that EI contributes to successful social adaptation (Martins et al., 2010; Punia and Sangwan, 2011; Davis and Rachel, 2016). However, EI may also contribute to negative emotional manipulation (Davis and Rachel, 2016). Today training and intervention of EI are of interest to scientists and the public, yet there is limited evidence of the underlying neural and hormonal mechanisms. Positive relationships were reported between EI and cognitive control (Checa and Fernández-Berrocal, 2015). Cognitive control can be influenced by sub-cortical emotional (bottom–up) processing, and this communication is mediated by testosterone and cortisol (Terburg et al., 2009). High testosterone was reported to down-regulate the interaction between cognitive and emotional systems and therefore diminishes the impact of cognitive control (Schutter and Van Honk, 2004). Basal cortisol also seems to correlate with cognitive control (Schutter and Van Honk, 2005). Few studies have took testosterone, cortisol and cognitive control processings as mechanisms underlying emotional intelligence, although there were some studies concerning other hormones and emotional perception in the context of emotional intelligence (Cardoso et al., 2014; Koven and Max, 2014; Milivojevic et al., 2014; do Vale et al., 2015). It is possible that the ratios of the basal levels of testosterone and cortisol are closely related to emotion regulation (Van Honk and Schutter, 2006). In this study we concentrate on the correlations among the hormones testosterone and cortisol, the cognitive control of emotion and EI in children.

Top–down modulation of emotional processing has been investigated as the cognitive control of emotion (Ochsner and Gross, 2005). According to the dimensional overlap theory (Kornblum et al., 1990, 1999), conflicts can be further categorized based on the overlap between the response (R), the task-relevant stimulus (SR), and the task-irrelevant stimulus (SI). The emotional flanker task contains the stimulus-stimulus (S-S) conflicts that SR (the target emotional facial expression) overlaps with SI (the bilateral distractor emotional faces) (Fenske and Eastwood, 2003; Liu et al., 2013). The emotional Stroop task contains both S-S and S-R conflicts, with the affective word (“FEARFUL” or “HAPPY”) on an emotional (happy or fearful) face, and participants are required to report the expression on the face (Etkin et al., 2006, 2010; Egner, 2008; Liu et al., 2010; Chechko et al., 2012; Soutschek and Schubert, 2013). It is reported that S-S and S-R conflicts involve distinct conflict control processes (Egner et al., 2007; Akcay and Hazeltine, 2011; Li et al., 2015) and rely on different neural substrates, since S-R conflict tasks activated the anterior cingulate cortex (ACC), precuneus and supplementary motor areas and S-S conflict tasks activated the inferior parietal cortex (Liu et al., 2004). The empirical study adopting color-object Stroop task to investigate the developmental changes of stimulus (S) interference and response (R) interference in 6–10 years old children found that the response interference control matured later (at age 10–12 years) than the stimulus interference control (at age 6–7 years), which further suggested the distinctive conflict control processes on the S-S and S-R conflicts from the child development evidences (Jongen and Jonkman, 2008).

Electrophysiological studies showed that conflict control processes are composed of two subprocessings: the N2 component of event-related potentials (ERPs), peaking at approximately 200 ms after stimulus onset, is associated with detection on both emotional (Shen et al., 2013; Fan et al., 2016; Xue et al., 2016) and non-emotional conflicts (Liu et al., 2010; Larson et al., 2014). The P3 and/or slow potential (SP) components, with a central-parietal neural distribution, are related to conflict resolution on both emotional (Fan et al., 2016; Xue et al., 2016) and non-emotional conflicts (Jonkman, 2006; Abundis-Gutiérrez et al., 2014). Both the cognitive control of emotional and non-emotional conflicts has been shown to activate the ACC, the dorsolateral prefrontal cortex (DLPFC), the parietal regions, the insula and the visual cortex (Chechko et al., 2012; Soutschek and Schubert, 2013).

Development of the interaction between emotional brain and cognitive brain could impact cognitive control of emotional processing throughout childhood and adolescence (Heller et al., 2016). The triple balance model of emotion (Van Honk and Schutter, 2006) hypothesized that the balance between the emotional brain and the cognitive brain would influence emotional processing at three levels which were linked to testosterone and cortisol. It has been reported that the first significant increase of testosterone occurs at 10 (SizoNenko and Paunier, 1975). Puberty peaks in brain functional activity showed to be related to testosterone (Braams et al., 2014). Previous studies demonstrated a possible negative association between testosterone levels and cortical response to word-face Stroop conflicts in 10- to 15-year-old adolescents (Cservenka et al., 2015), but during adults’ cognitive control of emotional processing, lower testosterone levels were associated with both stronger (Volman et al., 2011) and weaker response (Van Strien et al., 2009). There were also inconsistent results of the associations between the cortisol levels and executive functions (Raffington et al., 2018). Conflict detection might be influenced by testosterone and cortisol, since cortisol might enhance fear sensitivity (Watling and Bourne, 2013) and testosterone might enhance reward sensitivity (Van Honk and Schutter, 2006). According to the three balance model of emotion and the existing evidence, the testosterone/cortisol ratios can be related to cortical activities and behavioral tendencies in emotional processing (Terburg et al., 2009), but there are little evidence about the associations between testosterone/cortisol ratios and neural dynamic processing of emotional cognitive control.

To our knowledge, this is the first study to investigate the possible mediation effects of cognitive control of emotion on the relationships between hormones and EI components. The aims of the current study were to investigate the relationships among EI, the emotional conflict processing, and daily circulating testosterone and cortisol in 10- to 11-year-old children. We mainly focused on 10- to 11-year-old children in the current study for the following reasons. First, it is found that testosterone show their first significant rise at 10 years old and further lead the important influence on the brain reactivity (SizoNenko and Paunier, 1975; Nguyen et al., 2013). Second, most studies found that it is a crucial age for the neurodevelopment of cognitive control abilities (Waxer and Morton, 2011; Larson et al., 2012; Erb et al., 2017). Different types of conflict control develop at different speeds and with varied patterns (Jongen and Jonkman, 2008; Bryce et al., 2011) and may be indistinguishable in children up to 9 years of age, but may be related yet separable by 10–11 years of age (Brydges et al., 2014). Third, but not the least, at this age, the neural function of frontal and limbic areas gets more mature and starts to strengthen the interconnections to facilitate children’s perception and regulation on emotional information and states (Derntl et al., 2009; Sapienza et al., 2009; Stanton et al., 2011; Terburg et al., 2012). We adopted the emotional flanker task and emotional Stroop task to measure the emotional conflict control processing. We hypothesized that children’s high testosterone/cortisol ratios would diminish the efficiency of brain activities in emotional conflict control processes, and the efficiency of emotional conflict control processing would be positively associated with EI.

## Materials and Methods

### Participants

Thirty-six right-handed children participated in the experiment, and the data of two children were removed from analysis because of too much head movement. The data of remaining 34 children (16 girls [10.56 ± 0.32 years old] and 18 boys [10.90 ± 0.29 years old]) were further analyzed. The parents of child participants accomplished the written questionnaire, in which they were asked that whether the participant and his/her family had the neurological and/or psychiatric disorders. None of the participants reported that he/she or his/her family had neurological or psychiatric disorders. All the participants had normal or corrected-to-normal visual acuity, and they were naïve to the purpose of the experiment. This study was approved by the Ethics Committee of the Institute of Psychology, Chinese Academy of Sciences and School of Psychology, Capital Normal University. The work described has been carried out in accordance with the Code of Ethics of the World Medical Association (Declaration of Helsinki) for experiments involving humans and Uniform Requirements for manuscripts submitted to biomedical journals. The ERP, behavioral measurements and saliva collection were all undertaken with the understanding and written consent of the participants’ parents.

### Procedure

The brief outline of the time course of the tests: first, the salivary collection for all participants was on a school day before the ERP tasks; second, the completion of the emotional flanker and Stroop tasks with EEG recording; then, the completion of paper-pencil version of Emotional Intelligence Scale questionnaire (EIS).

### Salivary Collection and Analysis

Salivary testosterone and cortisol samples were collected within the same hours from all participants to minimize seasonal and diurnal variation as much as possible. Salivary collection was scheduled on a school day when no EEG recordings were scheduled. Participants were asked to rinse their mouths thoroughly 1 h before the saliva collection to avoid contamination from food debris and prevent sample dilution. During the interval between the mouth rinse and the saliva collection, the participants were asked to abstain from food or drink. Saliva samples were collected twice during a half-hour period from 8:40 a.m. to 9:10 a.m. Participants were instructed to pull the sponge out of the Salivette and place it into their mouth. They were told to chew the sponge very gently and roll it around in their mouth for 2 min. Saliva samples were then stored at −20°C as soon as possible. Testosterone and cortisol levels were determined using testosterone and cortisol ELISA kits (DRG, Germany). For testosterone, the reported inter- and intra-assay coefficients of variance were < 9.6 and 13.8%, respectively, with an analytical sensitivity of 1.9 pg/ml. For cortisol, the reported inter- and intra-assay coefficients of variance were < 7.5 and 4.5%, respectively, with an analytical sensitivity of 0.537 ng/mL.

### Emotional Flanker and Stroop Tasks

Each participant was instructed to participate in two computerized emotional conflict control tasks with EEG recordings, including the emotional flanker and the emotional Stroop task. The presentation sequence of these two tasks was counterbalanced among participants.

#### Emotional Flanker Task

The facial images in the revised emotional flanker task (Fenske and Eastwood, 2003; Liu et al., 2013) were of the face of six models (three males, three females) displaying both happy and fearful faces. The face stimuli were from our lab’s collection, and they were collected and used under the standardized procedure. Another 30 volunteers (male, 16, female, 14; age range 22.3–28.7 years) were instructed to assess the valence and arousal of each facial image by using normative nine-point scale. For the valence rating, happy images (Mean = 7.71, Standard deviation [SD] = 0.34) featured higher valence scores than fearful images (Mean = 2.11, *SD* = 0.31) (*p* < 0.001). For the arousal rating, there were no significance between happy (Mean = 6.82, *SD* = 0.35) and fearful images (Mean = 7.01, *SD* = 0.33) on arousal scores (*p* > 0.05). Each stimulus consisted of a central target face and two bilateral rows of two faces each, with the five faces in each stimulus from a single model. The visual angle of each stimulus was 1° vertical and 3.8° horizontal. According to the target facial expressions and the congruency between the target facial expression and the bilateral facial expressions, there were four types of trials: target fearful face in congruent trials (FFFFF), target fearful face in incongruent trials (HHFHH), target happy face in congruent trials (HHHHH), and target happy face in incongruent trials (FFHFF).

#### Emotional Stroop Task

The stimuli in the revised emotional Stroop task (Etkin et al., 2006) were the same with the emotional flanker task. Each stimulus consisted of a gray facial expression image overlaid with the red Chinese word “
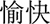
” (happy) or “
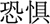
” (fearful). The visual angle was approximately 1° × 1.8°. Participants were instructed to concentrate on the facial expression and ignore the word over each presented stimulus. In the congruent condition, the facial expression was compatible with the meaning of the word. For the incongruent condition, the facial expression was incompatible with the meaning of the word.

**FIGURE 1:**
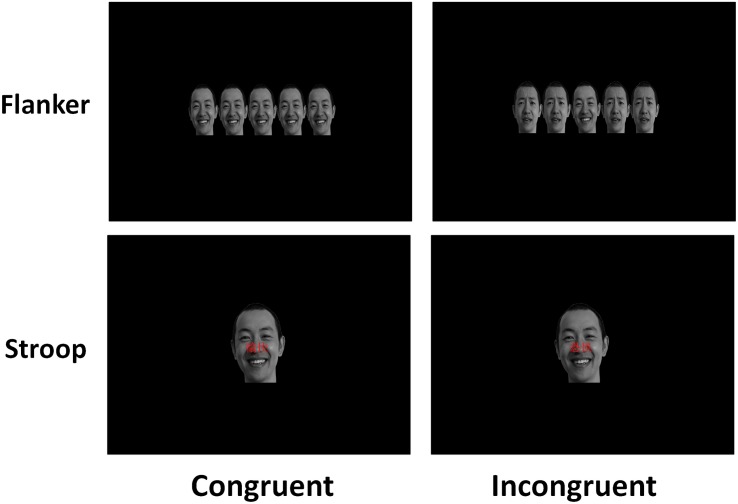
The sample stimuli and procedure in the emotional flanker task and the emotional Stroop task.

For these two tasks, all the stimuli were presented on a computer monitor with a black background (17 inches, 1024 × 768 at 100 Hz), and the participant’s viewing distance was 65 cm. Participants were required to press the left or right button to judge whether the facial expression was happy or fearful, and the mappings of the left and right index fingers to the happy and fearful stimuli were counterbalanced among participants. In both tasks, each trial began with a grey fixation cross “ + ” displayed for 250 ms and the presentation of a stimulus for 1500 ms, followed by randomly varied inter-trial intervals (ITIs) between 800 and 1000 ms. In the practice section, a total of 16 trials were displayed to allow participants to become familiar with the response rules, and the formal experiment section consisted of 4 blocks with 65 trials in each block. Each task consisted of 65 congruent trials with happy faces, 65 congruent trials with fearful faces, 65 incongruent trials with happy faces, and 65 incongruent trials with fearful faces. The participants were permitted to rest for 2–3 min after each block, and the whole task lasted approximately 18 min. In the two tasks in our study (see Figure 1), fearful conflict refers to cognitive conflict in trials with fearful target face. Happy conflict refers to cognitive conflict in trials with happy target face.

### EIS Questionnaire Measurement

Participants were required to complete the Chinese version of the EIS, which has been found to be suitable for measuring emotional intelligence of children and adolescents (Wang, 2002). EIS contains 33 items and is used to measure four EI abilities with the Emotion Perception (EP), Managing Self Emotions (MSE), Managing Others’ Emotions (MOS), and Emotional Utilization (EU) subscales (Wang, 2002; Sevdalis et al., 2007).

### EEG Recording and Data Analysis

The electroencephalogram (EEG) was recorded from sixty-four electrodes embedded in a Neuroscan cap with the electrodes placed according to the 10–20 system locations. Four bipolar electrodes were placed on the outer canthi of both eyes and the inferior and superior areas of the left eye to monitor the vertical and horizontal electrooculogram (VEOG and HEOG, respectively). The EEG signal with a nose reference was continuously recorded with online filters at 0.05–100 Hz and was amplified at a sampling rate of 1000 Hz. The electrode impedance was kept below 5 kΩ. The signal was epoched into trials with 100 ms prior to (for baseline correction) and 1000 ms after the stimulus onset, and epochs exceeding ± 100 μV at any electrode were excluded. The averaged ERPs were further digitally filtered off-line (zero phase shift; bandwidth: 1 and 30 Hz; slope: 24 dB/octave). The N2 and SP components were further analyzed according to previous literature (Larson et al., 2014) and current ERP data. The N2 components were analyzed over the fronto-central brain areas (average from F3, FC3, Fz, FCz, F4, and FC4) during the 220–370 ms time window after stimulus onset. The SP component was analyzed over the parieto-central areas (average from CP3, P3, CPz, Pz, CP4, and P4) in a time window of 510–680 ms.

For the behavioral and electrophysiological data analyses of the conflict control tasks, the response accuracy, reaction time (RT), and mean amplitudes and peak latencies of the N2 and SP components were analyzed with 2 × 2 × 2 × 2 repeated ANOVAs with within-subject independent variables (IVs) of Task (flanker, Stroop), Expression (happy, fearful) and Congruency (congruent, incongruent) and the between-subject IV of Gender (boy, girl). The Greenhouse–Geisser correction for violations of sphericity was used where appropriate, and the significant interactions were tested by Sidak test for multiple comparisons.

Consistent with other studies (Nguyen et al., 2013), a natural logarithm transformation of the levels of testosterone (ln_T) and cortisol (ln_C) and the T/C ratio (ln_ T/C) was used to avoid analytical bias. Since the Shapiro-Wilk test indicated that ln_T was non-normally distributed in the samples from the boys (*p* < 0.05), Spearman’s correlation analysis was used to examine the interrelationships among hormone levels, behavioral data and brain activities in boys and girls separately. Pearson correlation analysis was used to examine the non-sex-specific associations.

In addition, a mediation model was built to establish the roles of the neural activities during emotional conflict control processes in the correlation between the hormone levels and the EI abilities of individuals. According to Baron and Kenny’s (1986) conventions, the total effect was considered to be the association of the independent variable (IV) with the dependent variable (DV), the direct effect was the association of the IV with the DV after adjusting for the mediating variable (MV), and the indirect effect was path a (relation between IV and MV) × path b (relation between MV and DV after controlling for IV). The significance of the indirect effects was measured by a bootstrapping procedure (Preacher and Hayes, 2008). We used 10,000 samplings to generate 95% confidence intervals (CIs). If the CIs did not contain zero, then the association between the IV and DV was significantly explained by the MV (*p* < 0.05).

## Results

### Hormone Levels and Emotional Intelligence

There were no significant differences between boys and girls on the hormonal assay results (*p* > 0.05) and on EI scores results (*p* > 0.05) (Table 1).

**TABLE 1:** Descriptive statistics and associations between hormones and EIS scores.

			**Association with hormone (boys and girls together)**			
			
**Measure**	**Boys**	**Girls**	***EIS****-***EP**	***EIS****-***MSE**	***EIS****-***MOS**	***EIS****-***EU**
T(pg/ml)	20.70 (6.84)	20.44 (7.87)	0.03	−0.40^∗^	–0.14	–0.161
C(ng/ml)	2.83 (1.26)	2.86 (0.75)	–0.04	−0.42^∗^	–0.032	0.006
T/C ratio (× 10^–3^)	7.96 (2.77)	7.16 (1.99)	0.08	–0.17	–0.122	–0.190
Age (years)	10.90 (0.29)	10.56 (0.32)				
*EIS-EP*	37.16 (4.84)	36.56 (4.07)				
*EIS-* MSE	34.22 (6.74)	34.56 (3.28)				
*EIS-* MOS	31.11 (4.48)	31.40 (4.25)				
*EIS-* EU	15.67 (3.01)	15.58 (2.94)				

*Data are presented as mean and standard deviation. Total *N* = 34. Components in the EIS questionnaire: EP, Emotion Perception; MSE, Managing Self Emotions; MOS, Managing Others’ Emotions; EU, Emotional Utilization. ^∗^*p* < 0.05.*

### Behavioral Performances on the Two Tasks

For accuracy in the two tasks (Table 2), ANOVA showed a significant main effect of Gender (*F*_(__1_,_32__)_ = 13.1, *p* < 0.001, η^2^ = 0.29), with girls providing more accurate responses than boys. Congruency also showed a significant main effect (*F*_(__1_,_32__)_ = 31.2, *p* < 0.001, η^2^ = 0.49), with participants exhibiting a higher accuracy in congruent trials than incongruent trials.

**TABLE 2:** Mean and standard deviation of response accuracy and reaction time (RT) in the two tasks.

		**Flanker**		**Stroop**	
		**Congruent**	**Incongruent**	**Congruent**	**Incongruent**
Boys	Accuracy	0.83 (0.07)	0.81 (0.08)	0.88 (0.06)	0.77 (0.10)
	RT	695 (125)	703 (130)	706 (135)	746 (149)
Girls	Accuracy	0.92 (0.05)	0.88 (0.07)	0.95 (0.04)	0.85 (0.07)
	RT	688 (114)	704 (114)	723 (100)	803 (109)

In the analysis of the RT, a significant main effect of Task was observed (*F*_(__2_,_64__)_ = 10.5, *p* < 0.001, η^2^ = 0.25), with participants responding faster in the flanker task than in the Stroop task (*t*_(__32__)_ = −2.8, *p* < 0.05). The main effect of Expression was also significant (*F*_(__1_,_32__)_ = 9.5, *p* < 0.01, η^2^ = 0.23), and RTs were shorter in response to happy faces than to fearful faces. The interaction between Task and Congruency was detected (*F*_(__2_,_64__)_ = 23.9, *p* < 0.001, η^2^ = 0.43), and RTs were shorter in congruent trials than incongruent trials in the flanker (*t*_(__32__)_ = −3.7, *p* < 0.001) and the Stroop tasks (*t*_(__32__)_ = −8.2, *p* < 0.001).

### ERP Waveforms

**FIGURE 2:**
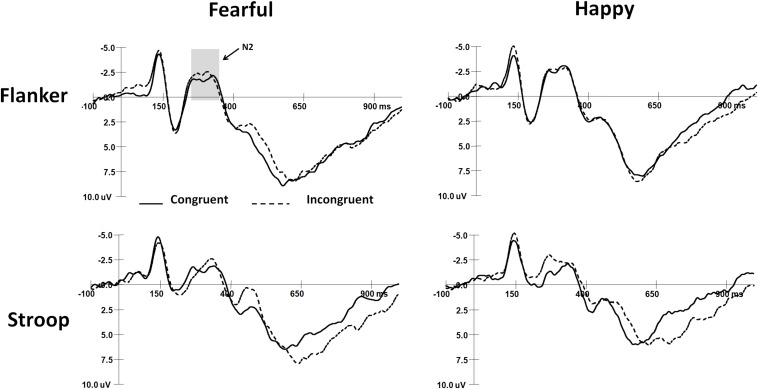
The grand-average waveforms of N2 components in two tasks.

**FIGURE 3:**
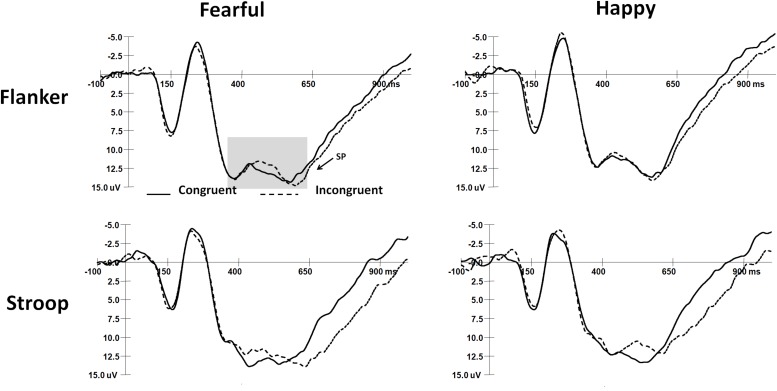
The grand-average waveforms of SP components in two tasks.

The mean peak latencies and amplitudes of N2 and SP components are shown in Table 3. The grand average waveforms of N2 and SP are displayed in Figures 2, 3, respectively.

**TABLE 3:** Mean peak latencies (ms) and amplitudes (μV) of N2 and SP components in the two tasks.

		**Flanker**		**Stroop**	
		**Congruent**	**Incongruent**	**Congruent**	**Incongruent**
Boys	N2 latency	284 (28)	281 (30)	290 (38)	293 (36)
	N2 amplitude	−4.84(4.61)	−5.59(4.3)	−3.57(3.6)	−3.74(3.99)
	SP latency	578 (40)	562 (32)	555 (33)	585 (34)
	SP amplitude	15.5 (8.55)	15.66 (8.22)	13.95 (8.45)	13.73 (7.44)
Girls	N2 latency	285 (33)	280 (31)	286 (33)	294 (33)
	N2 amplitude	−4.80(3.58)	−4.94(3.4)	−4.90(3.79)	−5.93(5.56)
	SP latency	584 (40)	587 (37)	565 (28)	598 (40)
	SP amplitude	14.56 (8.17)	14.98 (7.74)	13.4 (8.31)	13.96 (6.62)

#### N2 Components

For N2 amplitudes, the interaction among Task, Expression, and Gender was marginally significant (*F*_(__2_,_64__)_ = 3.0, *p* = 0.06), and boys exhibited more negative N2 responses to happy faces than fearful faces in the flanker task (*t*_(__64__)_ = 2.79, *p* < 0.01). There were no significant main effects or interaction effects for N2 latencies.

#### SP Components

SP latencies showed a significant main effect of Expression (*F*_(__1_,_32__)_ = 5.87, *p* < 0.05, η^2^ = 0.16), with happy faces inducing shorter SP latencies than fearful faces. A significant main effect of Congruency was also observed (*F*_(__1_,_32__)_ = 22.1, *p* < 0.001, η^2^ = 0.41), with shorter SP latencies in congruent trials than in incongruent trials. Additionally, the interaction among Task, Expression, and Congruency was significant (*F*_(__2_,_64__)_ = 4.4, *p* < 0.05, η^2^ = 0.12). In the flanker task, SP latencies were shorter in congruent trials than in incongruent trials when the target faces were fearful (*t*_(__32__)_ = 3.7, *p* < 0.001). In the Stroop task, SP latencies were shorter in congruent trials than in incongruent trials when faces were fearful (*t*_(__32__)_ = 3.7, *p* < 0.001) and happy (*t*_(__32__)_ = 3.6, *p* < 0.001). SP latencies were also shorter in response to happy faces than to fearful faces in incongruent trials of the flanker task (*t*_(__32__)_ = 3.5, *p* < 0.01). There were no significant main effects or interaction effects for SP amplitudes.

### The Correlations Among Emotional Abilities, Emotional Conflict Control, and Hormone Levels

For the whole sample, negative associations were found between testosterone and Managing Self Emotions (*r* = −0.40, *p* < 0.05), and between cortisol and Managing Self Emotions (*r* = −0.42, *p* < 0.05), and no significant correlations between brain activities and Managing Self Emotions (all *p* > 0.05) were found.

There were significant negative correlations between testosterone and accuracy (*r* = −0.36, *p* < 0.05), and between cortisol and accuracy (*r* = −0.36, *p* < 0.05) in the congruent trials in the emotional flanker task when target faces were fearful. There were also significant negative correlations between cortisol and accuracy (*r* = −0.45, *p* < 0.01) in the congruent trials in the emotional Stroop task when target faces were happy.

In the flanker task, N2 latencies in congruent trials were positively correlated with testosterone and cortisol levels when the target faces were fearful (testosterone: *r* = 0.46, *p* < 0.01; cortisol: *r* = 0.45, *p* < 0.01) (Table 4). T/C ratios were positively correlated with the differences in N2 latencies between incongruent and congruent trials in the Stroop task over the mid-line area (*r* = 0.37, *p* < 0.05). In the Stroop task, SP amplitudes were positively correlated with T/C ratios (see Table 4).

**TABLE 4:** Associations between brain activities and testosterone-cortisol balance.

	**Testosterone**	**Cortisol**	**T/C ratio**
**Flanker**			
N2 latency_left_fearful^1^	0.46^∗∗^	0.45^∗∗^	–0.01
^D^N2 latency_left_fearful	−0.37^∗^	−0.40^∗^	0.05
^D^N2 latency_middle_fearful	–0.22	−0.37^∗^	0.12
**Stroop**			
^D^N2 latency_middle_fearful	–0.01	–0.26	0.37^∗^
^D^N2 amplitude_left_happy	–0.23	−0.36^∗^	0.18
**Stroop**			
SP latency_left_fearful^1^	–0.15	0.07	−0.35^∗^
SP amplitude_middle_fearful^2^	–0.09	−0.40^∗^	0.38^∗^
SP amplitude_middle_happy^1^	–0.13	–0.45^∗∗^	0.39^∗^
SP amplitude_middle_happy^2^	–0.16	–0.60^∗∗^	0.53^∗∗^
SP amplitude_right_fearful^2^	–0.21	–0.49^∗∗^	0.37^∗^
SP amplitude_right_happy^1^	–0.17	–0.51^∗∗^	0.42^∗^
SP amplitude_right_happy^2^	–0.21	–0.59^∗∗^	0.46^∗∗^
SP amplitude_overall_fearful^2^	–0.15	−0.43^∗^	0.35^∗^
SP amplitude_overall_happy^1^	–0.20	–0.53^∗∗^	0.40^∗^

*^*D*^Congruency effects; ^1^Congruent trials; ^2^Incongruent trials. ^∗^*p* < 0.05, ^∗∗^*p* < 0.01.*

#### Girls

Emotional Utilization scores in EI negatively correlated with cortisol levels (*r* = −0.57, *p* < 0.05). Cortisol levels were also associated with the SP amplitude in incongruent trials of the flanker task (*r* = 0.63, *p* < 0.01) and the SP amplitude difference (*r* = 0.51, *p* < 0.01) when the target face was fearful. Hormone levels were negatively correlated with accuracy in congruent trials of the flanker task when the facial expression was fearful (*r* = −0.52, *p* < 0.05, testosterone; *r* = −0.68, *p* < 0.05, cortisol). Cortisol levels were positively correlated with RT in the incongruent trials of the Stroop task when the facial expression was fearful (*r* = 0.51, *p* < 0.05), and RT in the congruent trials of the Stroop task when the facial expression was happy (*r* = 0.50, *p* < 0.05). Cortisol levels correlated with accuracy in congruent trials of the Stroop task when the facial expression was fearful (*r* = −0.56, *p* < 0.05) and happy (*r* = −0.56, *p* < 0.05) and SP amplitude in incongruent trials of the flanker task when the target face was fearful (*r* = 0.54, *p* < 0.05). Finally, cortisol levels also correlated with SP amplitude differences when the facial expression was happy in the Stroop task (*r* = −0.51, *p* < 0.01).

#### Boys

Managing Self Emotions in EI was positively correlated with accuracy in the incongruent trials of the Stroop task (*r* = 0.55, *p* < 0.05). Emotion Utilization positively correlated with the SP amplitude difference in the flanker task when the target face was fearful (*r* = 0.48, *p* < 0.05). Testosterone levels were negatively correlated with Managing Self Emotions in EI (*r* = −0.51, *p* < 0.05). Testosterone levels were also negatively correlated with response accuracy (*r* = −0.51, *p* < 0.05) and positively correlated with N2 latency (*r* = 0.49, *p* < 0.05) in incongruent trials of the flanker task when the target face was happy. Testosterone levels were also negatively correlated with the difference in accuracy between congruent trials and incongruent trials of the Stroop task when the facial expression was fearful (*r* = −0.50, *p* < 0.05).

### The Mediation Effects Among Emotional Abilities, Emotional Conflict Control, and Hormone Levels

Based on the correlation results, we further assessed a mediation model including brain activity. We included the scores associated with Emotional Utilization of the girls as the DV, their cortisol levels as the IV, and the SP amplitudes in incongruent trials of the flanker task when the target face was fearful as the MV. The SP amplitude in incongruent trials of the flanker task when the target face was fearful mediated the influence of the cortisol levels on the utility of emotion in EI [95% *CI* = (−8.64, −0.54), *p* < 0.05].

## Discussion

To our best knowledge, this is the first study investigating the correlations among hormones (testosterone and cortisol), EI and neuronal activities during emotional conflict control processes in preadolescent children. Overall, the results suggested a more complicated picture of relationships than we expected. Although there were no significant correlations between hormone ratios and EI, lower hormone (testosterone or cortisol) levels were found to be related with better abilities of managing self emotions (a component of EI) in preadolescent children. Furthermore, we also found that the associations between cortisol and emotional utilization (another component of EI) were mediated by neural activities in conflict resolution on emotional conflicts in girls. Another finding was that T/C ratio correlated with conflict processing when processing fearful faces.

The comparison of different conflict control processes with varied conflict types in our study supported the dimensional overlap model (Liu et al., 2010) and further indicated that the varied combination of different dimensions of stimulus-response mappings may induce varied conflict control processes (Liu et al., 2004; Egner et al., 2007).

The current behavioral conflict control processes were not modulated by the valence of the target facial expression, which was inconsistent with previous findings in adults, potentially revealing that the brain functions of preadolescent children might not be sufficiently mature to support the interplay between these two processes. Our study revealed distinct response patterns dependent on emotional prosody. RTs were shorter in response to happy faces than fearful faces, which is consistent with previous evidence showing that happy expression was the first expression that children could identify (Watling and Bourne, 2013).

Testosterone levels may mediate cognitive function through attentional control processes (Martin et al., 2009). The current study showed that emotional conflict control processes in both boys and girls were associated with testosterone. For boys, lower testosterone levels were related to greater accuracy in conflict control tasks with happy conflicts (in the flanker task) and fearful conflicts (in the Stroop task) and were associated with shorter frontal responses to happy conflicts during conflict detection processing (in the flanker task). For girls, lower testosterone levels correlated with greater accuracy in congruent trials of the flanker task when the target faces were fearful, consistent with the findings of Van Strien et al. (2009). Similarly, for behavioral responses, Tyborowska et al. (2016) found that lower testosterone levels were associated with faster responses to happy faces than to angry faces; however, they also found that lower testosterone levels were associated with more errors with angry faces than with happy faces. They analyzed the behavioral data with boys and girls together. Previous studies have provided puzzling results upon examination of brain activities during emotional control. Cservenka et al. (2015) adopted an emotional Stroop task to study the effects of hormones on emotional conflict control processes and brain activity in adolescence and found significant gender effects, as testosterone was negatively correlated with frontal and striatal activities in male adolescents and cerebellar and precuneus activities in female adolescents. These findings revealed that testosterone could play important roles during emotional cognitive control processes and indicated that sex differences should be further examined.

Previous studies showed that children’s salivary cortisol levels were tightly associated with their inhibition control abilities and fear perception (Klimes-Dougan et al., 2001; Gunnar et al., 2009). Our present findings revealed a tight relationship between cortisol levels and emotional conflict control processes in both boys and girls. Upon examination of the neuronal activity during conflict resolution, we found that lower cortisol levels in girls were associated with better conflict resolution in the parietal cortex (greater SP amplitude) in fearful conflicts in the flanker task. Consistently, Schutter et al. (2002) revealed cortisol-related reductions in transmission between the left prefrontal and right parietal cortex in healthy subjects aged 20–28 years.

Interestingly, the relationships between hormones and brain activities in the flanker task showed that it might take longer to detect whether there were conflicts in the flanker task when all the five faces were fearful (congruent trials) when children bore higher hormone levels. At the same time, higher hormone levels correlated with smaller congruency effects of N2 latency when the target face was fearful. We speculated that these hormone-latency associations mainly indicated high concentration of hormone was related to the delay of processing fearful faces in congruent trials in the flanker task. The reason was that the congruency effects in N2 latency were outcomes of subtracting latency of congruent trials from latency of incongruent trials, However, there might be distinct brain mechanisms for the association between fearful expression processing and testosterone (Van Honk and Schutter, 2006) and cortisol (Watling and Bourne, 2013). Furthermore, a recent study reported that higher daily cortisol concentrations inhibited functional connectivity between the prefrontal cortex and the amygdala when processing fearful faces compared to neutral faces (Hakamata et al., 2017).

In addition, our findings might also indirectly indicate the association between fearful face processing and T/C ratio. This is the first study providing evidence for T/C ratios and brain activities in emotional processing in children. When faces were fearful, T/C ratios were correlated with SP latency and congruency effects on N2 latency, while neither testosterone or cortisol levels were correlated with them. This is similar with a previous study. Glenn et al. (2011) found that psychopathy was associated with an increased T/C ratio, but was not associated with testosterone or cortisol independently. In addition, the faster SP responses related to higher T/C ratios might indicate T/C-related behavioral impulsivity (Terburg et al., 2009; Romero-Martínez et al., 2016; Manigault et al., 2019). A recent study also found that high testosterone relative to cortisol, was associated with aggressive behavior in 16-year-old adolescents (Platje et al., 2015). However, since T/C ratio is not correlated with any component of EI, further studies should be carried out with larger sample size and different measures.

The lack of sex differences in testosterone levels, as reported in our results, is consistent with previous studies of children (Quaiser-Pohl et al., 2016; Nguyen et al., 2018). Distinguishing between the biologically active free fraction of gonadal hormone levels (as measured in saliva) and the amount of available hormone levels in total (as measured in blood) might be important (Quaiser-Pohl et al., 2016). As for cortisol levels, it has been proposed that sex-related differences in HPA regulation emerge at puberty (Wang et al., 2018).

Previous studies of adults found that EI is positively associated with affective executive function processes (Sevdalis et al., 2007). Our findings implied that different aspects of EI were related to varied neuronal activities in different brain areas during conflict control processes. Self-management of emotion was associated with both frontal and parietal activities during neuronal processes of conflict monitoring and conflict resolution of affective conflicts. However, emotional utilization was only associated with parietal activities during conflict resolution of affective conflicts.

We found that lower testosterone levels in boys were associated with better self-management of emotion in EI, and lower cortisol levels in girls were associated with better emotional utilization in EI. Bechtoldt and Schneider (2016) found that the testosterone and cortisol levels in male adults did not correlate with EI. One reason for this difference may be due to differences in the collection times of the saliva samples. Samples collected between 3 p.m. and 8 p.m. have been shown to contain relatively lower levels of testosterone and cortisol during the daytime (Nelis et al., 2009), whereas samples collected around 9 a.m., as in the current study, contain relatively higher levels. Another possible reason for the discrepancy is the criterion validity of the EI scale. Bechtoldt and Schneider (2016) specifically stressed that the ability-based tests of EI that they chose assessed emotion management by evaluating maximum performance measures in hypothetical contexts, which may be inadequate for predicting emotion-regulating behavior in real contexts. A significant mediation effect of emotional conflict control on the association between EI and hormones was found in girls. Lower cortisol levels were associated with better utilization of emotion in girls, and the association was mediated by a stronger parietal response to emotional conflicts. Our findings revealed an essential role of neural activities conflict resolution in mediating the hormonal effects on emotional abilities in girls.

There were several limitations in the current study. First, a single-point measure of hormones is a significant weakness of our study, and it would be better to measure salivary hormones on two or more consecutive days with multiple time-points on each day. Second, the small sample size is also a weakness; therefore, we adopted strict Bonferroni corrections for multiple comparisons. Third, the emotional words used in the current study may be not that emotional in terms of the emotion-inducing effect compared with facial expression images, and further studies should adopt some emotional words with higher arousal levels.

## Conclusion

The study shows that testosterone correlates with conflict detection and managing self emotions, while cortisol correlates with conflict detection and resolution as well as managing self emotions. Besides, in girls, neural activities during conflict resolution in the S-S conflicts mediate the correlation between cortisol levels and emotional utilization. In addition, the relationships between hormones and neural activities vary depending on the type of emotional conflict control task and emotional stimuli. We thereby provide supportive preliminary evidence for hormonal and neural mechanisms underlying emotional intelligence in preadolescence. Future studies can further investigate the involvement of hormone-mediated emotional processing during the development of emotional intelligence in children and adolescents, and latent state-trait modeling could be applied to model individual differences in salivary testosterone, cortisol, and their interaction in the future studies.

## Data Availability Statement

The datasets generated for this study are available on request to the corresponding author.

## Ethics Statement

The studies involving human participants were reviewed and approved by the Ethics Committee of the Institute of Psychology, Chinese Academy of Sciences and School of Psychology, Capital Normal University. Written informed consent to participate in this study was provided by the participants’ legal guardian/next of kin. Written informed consent was obtained from the individual(s) for the publication of any potentially identifiable images or data included in this article.

## Author Contributions

TL and FS wrote the first draft of the manuscript together and designed the study and interpreted the results, then revised the draft. DL undertook the EEG data processing and statistical analysis. JS supervised the project and revised the draft. All authors contributed to and have approved the final manuscript.

## Conflict of Interest

The authors declare that the research was conducted in the absence of any commercial or financial relationships that could be construed as a potential conflict of interest.
